# Supplementary figures and images for: From Trypomastigotes to Trypomastigotes: Analyzing the One-Way Intracellular Journey of Trypanosoma cruzi by Ultrastructure Expansion Microscopy
Source: Pathogens. 2024 Oct 2;13(10):866. doi: 10.3390/pathogens13100866 (PMC11510640; doi:10.3390/pathogens13100866)

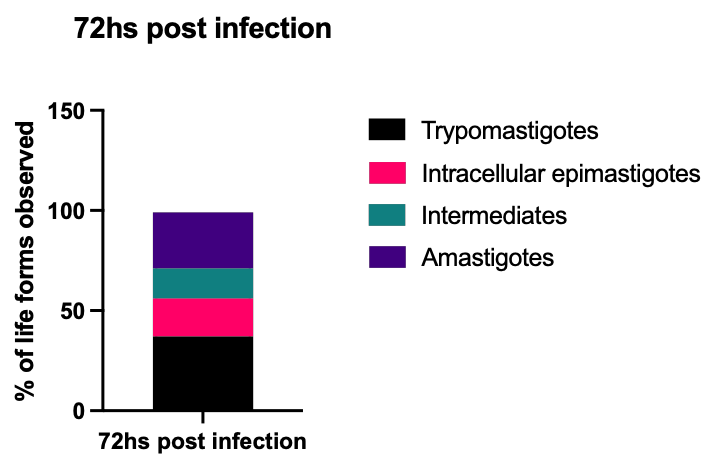

Supplement: Supplementary file 1 [file pathogens-13-00866-s001.zip › Suplementaries/SupFig5.png]

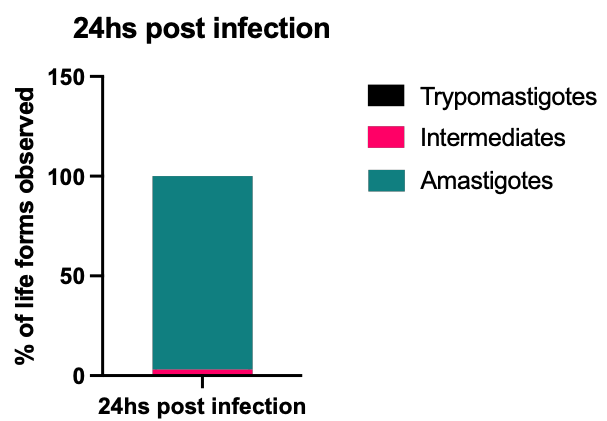

Supplement: Supplementary file 1 [file pathogens-13-00866-s001.zip › Suplementaries/SupFig4.png]

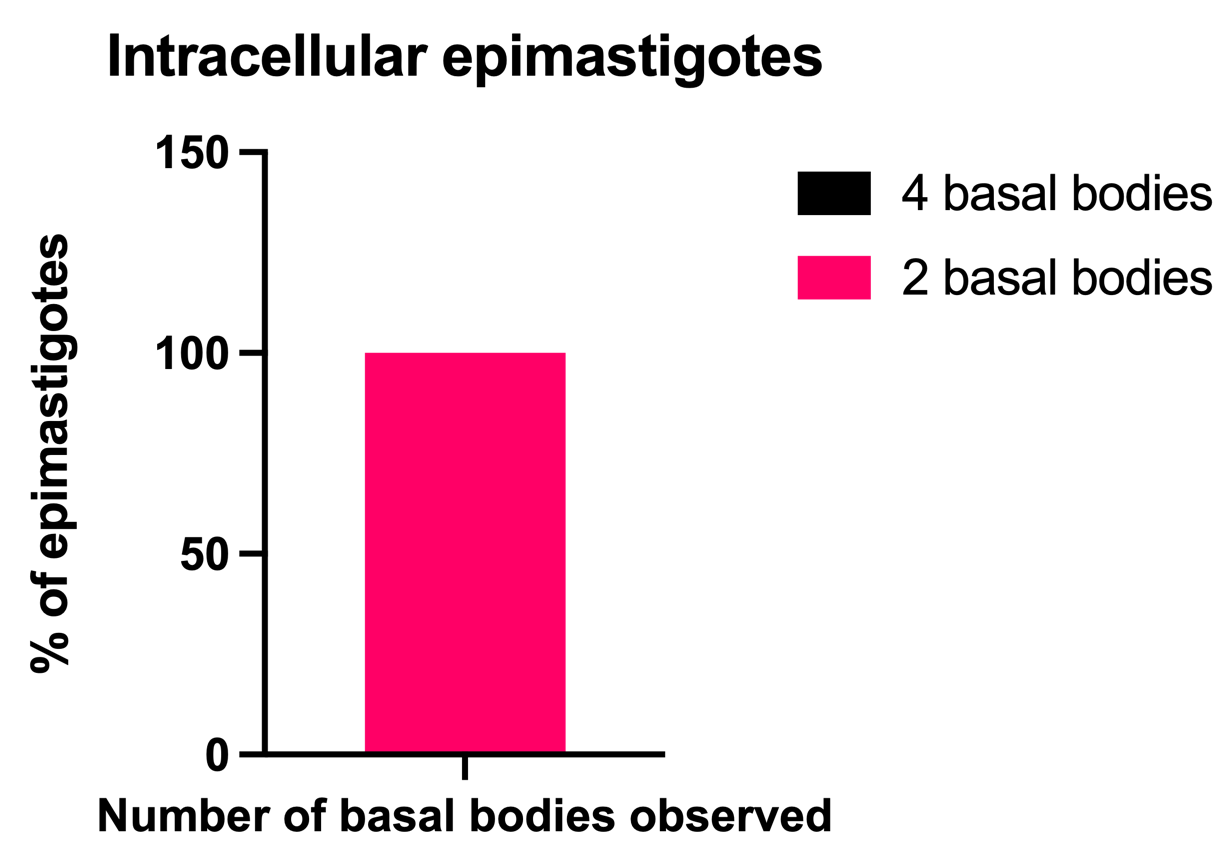

Supplement: Supplementary file 1 [file pathogens-13-00866-s001.zip › Suplementaries/SupFig6.png]

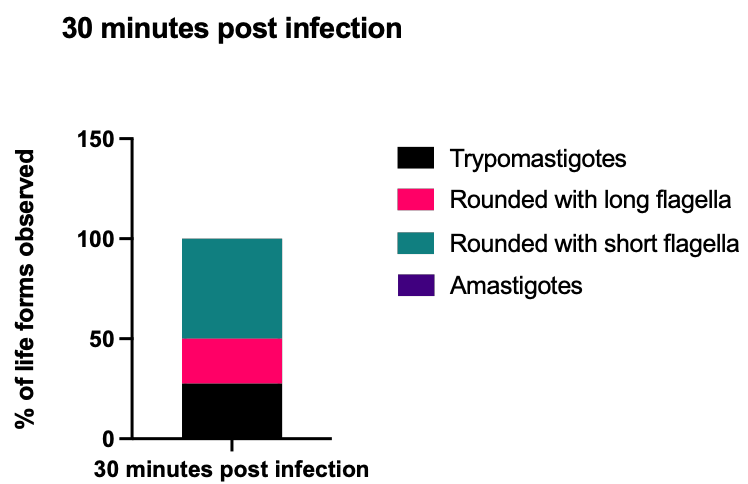

Supplement: Supplementary file 1 [file pathogens-13-00866-s001.zip › Suplementaries/SupFig3.png]

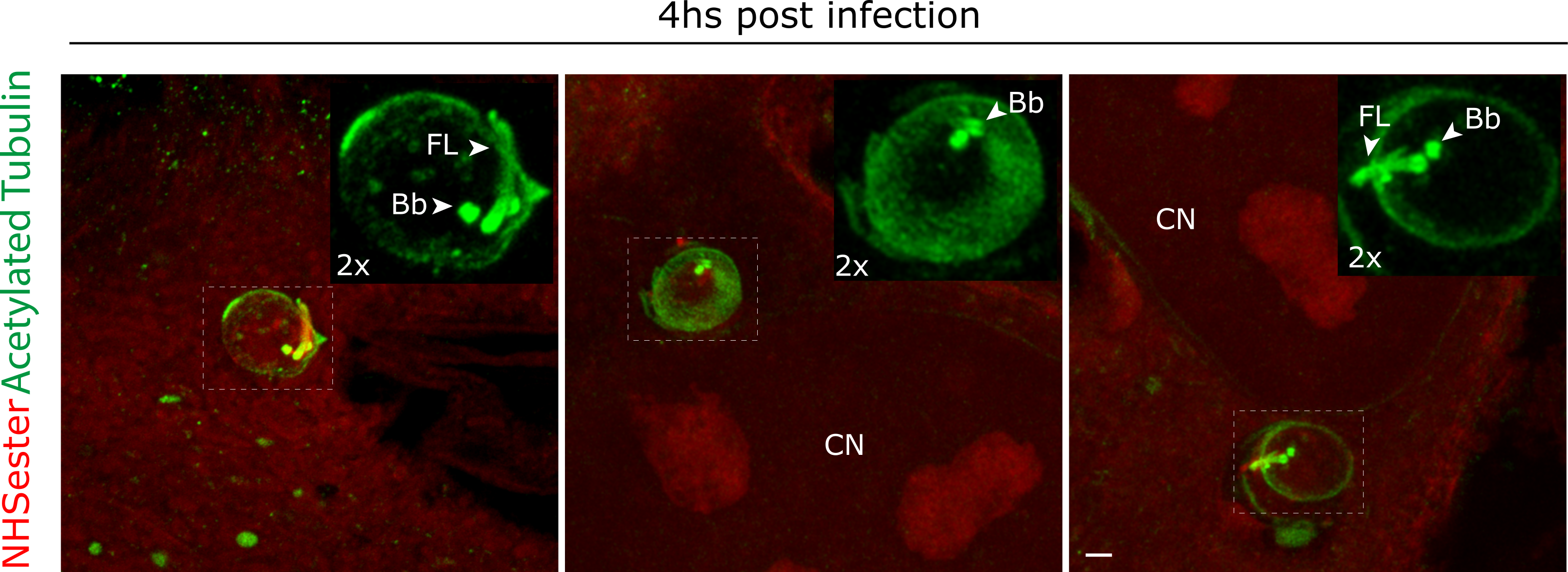

Supplement: Supplementary file 1 [file pathogens-13-00866-s001.zip › Suplementaries/supFig2.png]

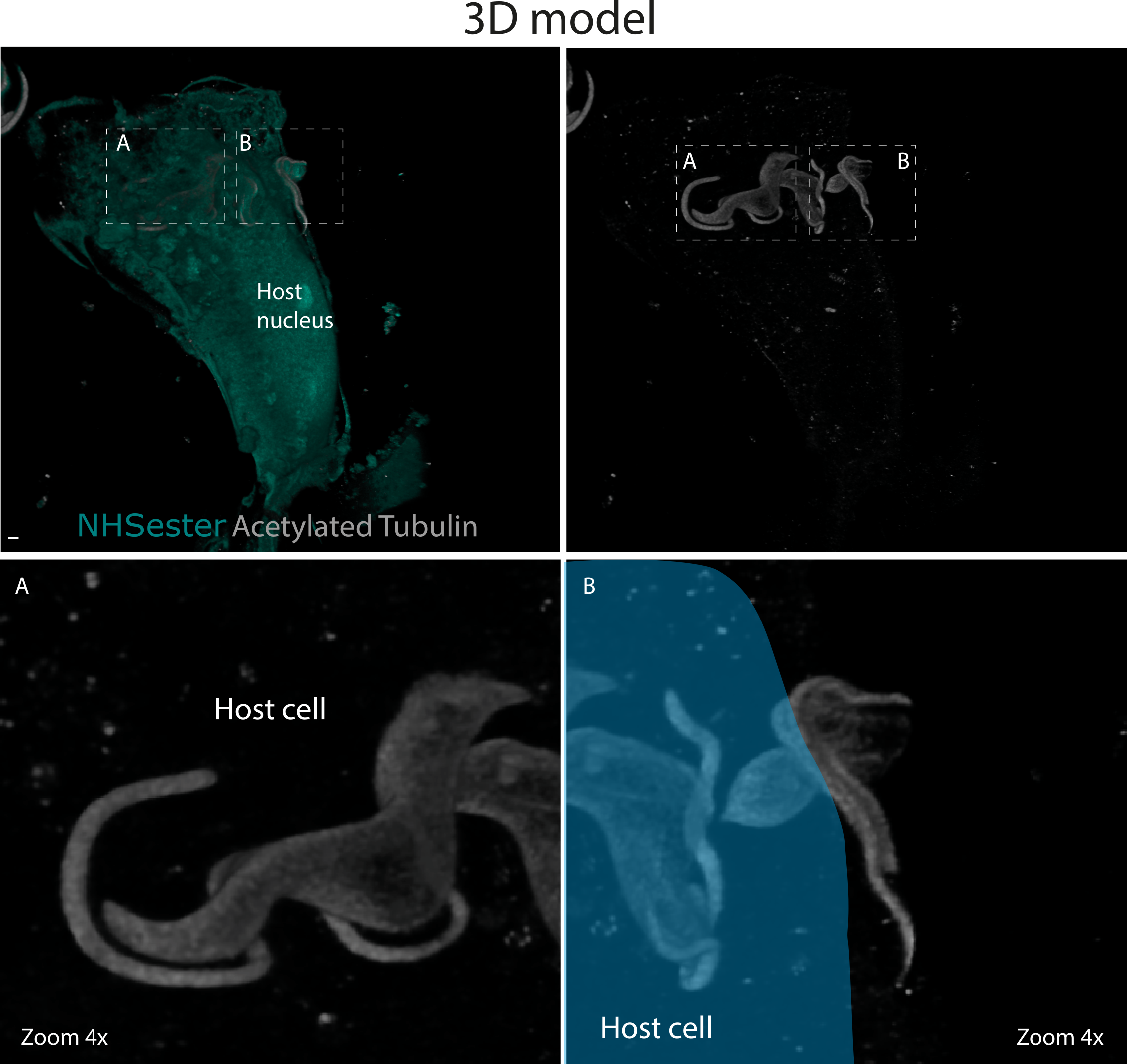

Supplement: Supplementary file 1 [file pathogens-13-00866-s001.zip › Suplementaries/SupFig1.png]
